# Qualitative Evaluation of the Effects of Professional Oral Hygiene Instruments on Prosthetic Ceramic Surfaces

**DOI:** 10.3390/ma15010021

**Published:** 2021-12-21

**Authors:** Francesco Grande, Edoardo Mochi Zamperoli, Mario Cesare Pozzan, Fabio Tesini, Santo Catapano

**Affiliations:** 1 Department of Surgical Sciences, CIR Dental School, University of Turin, 10126 Turin, Italy; 2 Department of Prosthodontics, University of Ferrara, Via Luigi Borsari 46, 44121 Ferrara, Italy; mochizamperolie@gmail.com (E.M.Z.); mariocesare.pozzan@edu.unife.it (M.C.P.); fabio.tesini@edu.unife.it (F.T.); cts@unife.it (S.C.)

**Keywords:** lithium disilicate, CAD-CAM zirconium, professional hygiene, qualitative analysis

## Abstract

During professional hygiene procedures, different instruments used may cause various damage to dental prostheses. Deplaquing and scaling with curettes and ultrasonic instruments may inadvertently increase the surface roughness of the material and the risk of future bacterial adhesion and/or also compromise the marginal seal of the prosthesis. Hence, the aim of this study was to assess the qualitative effects of two types of curettes and one piezoelectric instrument with a stainless-steel tip on three types of metal-free samples. After treating the samples with different instrumentations, they were analyzed using the scanning electron microscope and then underwent a qualitative microanalysis by using a spectroscopy machine. All the materials tested in this study have undergone significant changes of their superficial structure after instrumentation both with mechanical and manual instruments. Plastic curettes appeared to be less aggressive than the other instruments. Disilicate samples show a significantly lower degree of surface glazing erosion compared to the zirconia sample with all the instruments used.

## 1. Introduction

Today, several metal-free prostheses have become available and popular in prosthetic dentistry; they allow a better reproduction of the natural tooth, a higher aesthetics and require a lower preparation than metal-ceramic restorations avoiding some of their shortcomings [1]. Metal-free restorations proved their success both in the anterior [2] and posterior teeth [3,4] and thanks to the introduction of high-strength ceramic materials, they have become an option even in longer-span fixed dental prostheses [5,6]. In addition, with these materials, workflow could be completely digital, from the oral impression to the fabrication of the restorations [7,8]. Zirconia can be produced only using CAD-CAM systems by milling a presinterized block, while lithium disilicate could be fabricated both by milling machines and pressed fabrication following the lost wax technique [9,10]. In this way, the dental technician can fabricate restorations in lithium disilicate by using either the CAD design or after manual waxing procedures. Then, today lithium disilicate, as zirconia materials, could be milled from a prefabricated industrial block by sending the file of the design of the restoration to a laboratory or industrial milling machine. In this way, a full digital workflow can be performed without any physical model (if the impression is also digital). Alternatively, an additive manual waxing of the physical gypsum model of the patient had to be carried out before the conventional fabrication technique of fused pressed injection of disilicate. These two methods of fabrication result in different mechanical properties of the same material; hence, differences in surface qualitative evaluation after scaling could be interesting to carry out.

Oral hygiene procedures are necessary to reduce risks of gingival and periodontal diseases and then to promote the long-term success of prosthetic restorations [11,12,13]. During deplaquing and scaling, different instruments such as hand instruments, mechanical instruments, and ultrasonic scalers have been demonstrated to be effective for removal of plaque when using for several minutes [14,15]. The literature shows that treating a natural tooth with manual instruments takes from six to eight minutes (4–6 min for mechanical tools) [16,17]. Hence, during professional oral maintenance of patients rehabilitated with metal-free prostheses, attention must be paid. Iatrogenic surface damages of these prosthetic restorations especially at the cervical area may result in plaque retention and subsequent inflammation of surrounding soft tissues [18]. It is possible that professional plaque removal can inadvertently increase the risk of future bacterial adhesion [19] and/or also compromise the marginal seal. In fact, a smooth surface is preferred for optimal biocompatibility of restorative materials because rough surfaces are reported to encourage plaque retention and to mechanically irritate surrounding soft tissues [20].

Several types of curettes and scalers have been developed for professional oral hygiene procedures from universal to specific scaler, curettes and piezoelectric instruments fabricated with different materials. Several studies were performed to analyze the surface roughness of these materials in combination with different instruments [19,21]. 

The aim of this in vitro study was to assess the qualitative effects of professional hygiene maintenance of two types of curettes and one piezoelectric instrument with a stainless-steel tip on metal-free samples using the scanning electron microscope (SEM). In addition, qualitative micro-analyzes were carried out to highlight potential exchanges of material between the instruments used and the samples.

## 2. Materials and Methods

**Figure 1:**
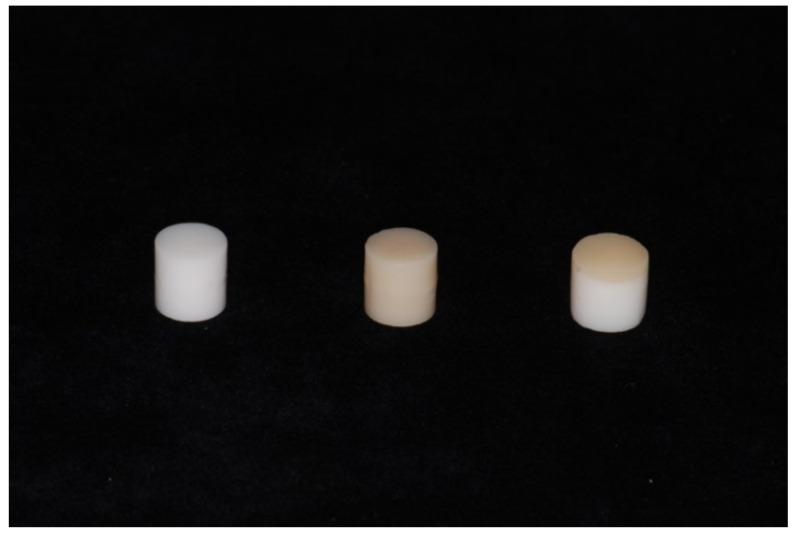
Metal-free samples.

Three cylindrical metal-free samples (Figure 1) of 1 cm in height and 1 cm in diameter were used. The samples were composed, respectively, of CAD-CAM milled zirconia (3D Pro-zir, Aidite, Santa Fe Springs, CA, USA), CAD-CAM milled lithium disilicate (IPS e.max CAD, Ivoclar Vivadent, Schaan, Liechtenstein) and high fusion pressed lithium disilicate (IPS e.max Press, Ivoclar Vivadent, Schaan, Liechtenstein). The three milled samples were produced from a CAD design, while the fusion pressed lithium disilicate samples were fabricated using a lost-wax casting technique. The zirconia samples were then sintered, and all the samples were sandblasted with aluminum oxide (100 um particles) and glazed (Ivocolor glaze paste, Ivoclar Vivadent IPS, Schaan, Liechtenstein) with a standardized technique and procedure. The technical procedures were performed by a 20 years experienced technician.

For each sample four distinct areas were marked and named A, B, C and D. 

In “A zones”, no instrumentation was performed, representing the “untreated control” surface that will serve as a reference. In “B zones”, the samples were treated with plastic curettes (Implacare™ II LG1/2 Tips with Handle Kit Curette; Hu-Friedy, Chicago, IL, USA). In “C zones”, the samples were treated with a steel curette (1/2 Langer Mini Five Curette; Hu-Friedy, Chicago, IL, USA). In “D zones”, the samples received a piezoelectric ultrasonic scaler treatment with a steel tip (Piezosteril 5 #C1 tip; Castellini, Bologna, Italy). A new instrument for each sample was used to avoid dullness problems.

**Figure 2:**
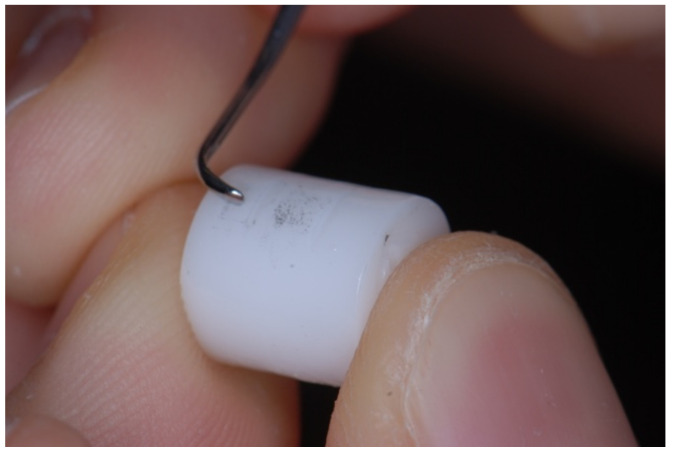
Examples of curettes instrumentation.

The instruments were used as recommended by the manufacturers. The samples were oriented horizontally in relation to the operator and kept still during scaling procedure (Figure 2). 

The untreated surface of each specimen was placed at the opposite side of scaling. Subsequential horizontal and oblique scaling motions were performed for each surface treated with curettes. The blades were oriented at a 70-degree angle to the ceramic surfaces with pressure that simulated clinical procedures [21], while the scaling tips were angled at approximately 15 degrees [22].

The time of treatment was established based on the study of Torfason and Badersten [14,15], who show that treating a natural tooth with manual instruments takes from six to eight minutes (4–6 min for mechanical tools). Assuming a supra- or iuxta-gingival instrumentation performed only on one face of the tooth, one minute of manual instrumentation was established for each treated area of the samples. 

The ultrasonic scalers were used for 30 s with a water-cooling system, using short and constant movements in the same direction, as described in previous studies [22]. 

All instrumentation was performed by the same experienced dental operator and in standard conditions.

After treatment, the samples were sent to the Center for Electron Microscopy of the University of Ferrara. To visualize surface scraping areas, a scanning electron microscope (SEM Zeiss Evo 40; Germany) was used, while to identify the chemical elements on the surfaces, a qualitative microanalysis by using a spectroscopy machine (EDS, Energy TEM INCA 300, Oxford Instruments, Buckinghamshire, UK) was carried out. EDS sensors allow a quick acquisition of the complete emission spectrum and the identification of the different components of the sample and their proportions in a relatively short time. Before the scan, the specimens were mounted on specific fixed supports and coated with a thin layer of graphite, a conductive material able to allow the scanning electron microscopy (SEM) without interfering with the qualitative analysis. 

For all the areas of each sample, SEM scans were performed at two different magnification levels (MAG = 100×; MAG = 550×) and a qualitative microanalysis was performed.

## 3. Results and Discussion

**Figure 3:**
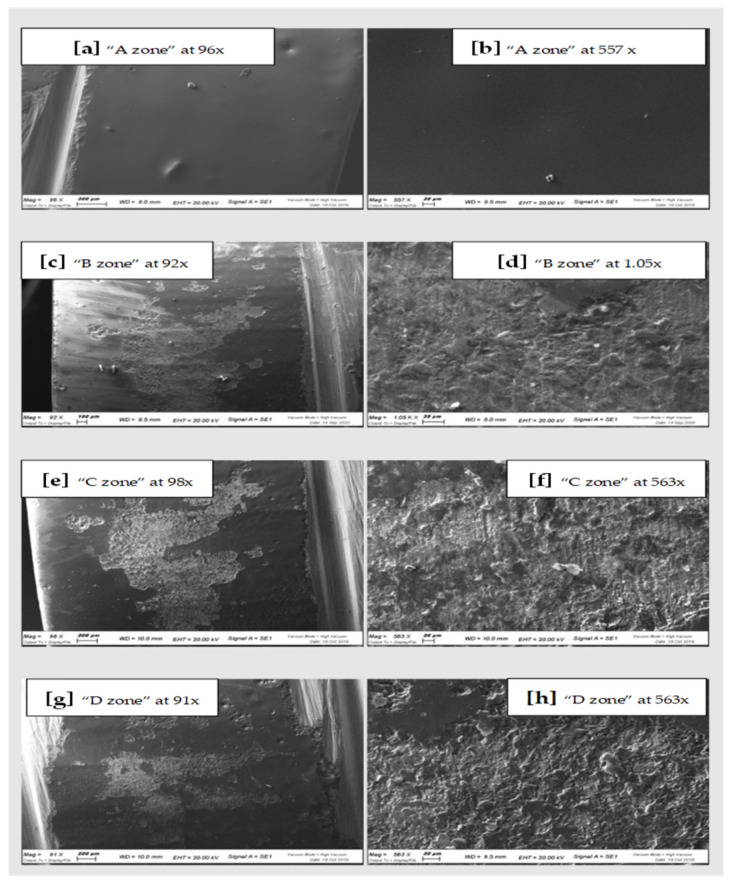
SEM scans of CAD-CAM Zirconia “A zone” (**a**,**b**), “B zone” (**c**,**d**); “C zone” (**e**,**f**) and “D zone” (**g**,**h**).

**Figure 4:**
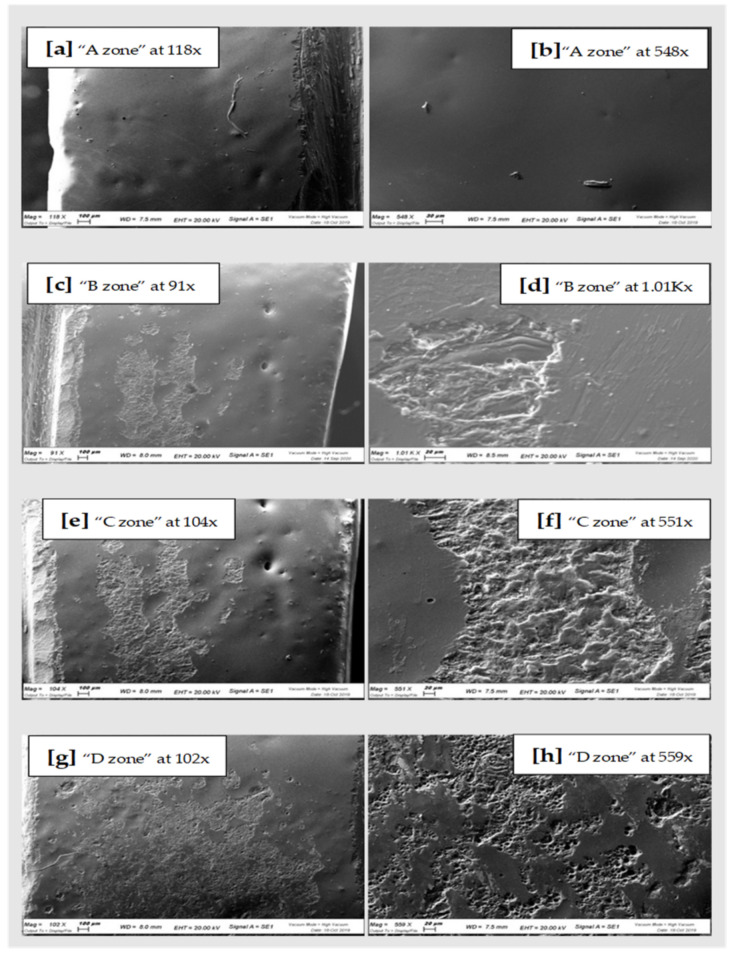
On the right SEM scans of pressed Lithium Disilicate “A zone” (**a**,**b**), “B zone” (**c**,**d**); “C zone” (**e**,**f**) and “D zone” (**g**,**h**).

**Figure 5:**
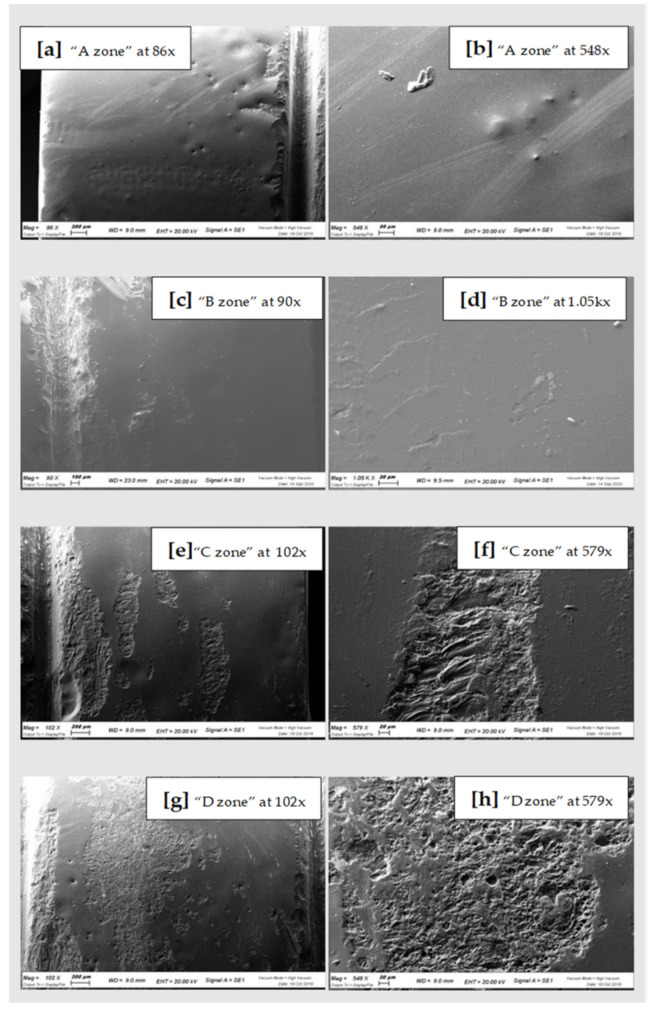
SEM scans of CAD-CAM Disilicate “A zone” (**a**,**b**), “B zone” (**c**,**d**); “C zone” (**e**,**f**) and “D zone” (**g**,**h**).

All the samples of the study were analyzed under a scanning microscope and then with the spectroscopy. The images of the scans at differentiated magnification are shown below (Figure 3, Figure 4 and Figure 5). Results of qualitative microanalyses are shown in Table 1, Table 2 and Table 3.

In general, comparing the different images at SEM by visual inspection, both the steel curette (Group C) and ultrasound steel tips (Group D) have visibly furrowed and/or scratched the structural surface of these materials, while plastic curettes (Group B) appeared to be less aggressive than the other instruments. Steel curettes (Group C) ere demonstrated to be more aggressive than the sonic driven (Group D) across all the samples. These findings could be justified by the different composition of the instruments (plastic vs steel) and from the sharpness, which is more pronounced for curettes than for the tips of piezoelectric instrument. 

Further observations provided evidence that steel curettes (Group C) caused superficial structure loss on all the materials tested (Figure 3e,f, Figure 4e,f and Figure 5e,f), with no evident visual differences between the different samples. Also, piezoelectric instrumentation (Group D) showed aggression on all the materials tested (Figure 3g,h, Figure 4g,h and Figure 5g,h); however, pressed disilicate demonstrated a higher resistance to scratching against mechanical instrumentation, maintaining intact many areas of its surface (Figure 4c–h). This is probably due to the different wear coefficient of the pressed disilicate material [23]. It is also interesting to highlight those processes such as delamination, plastic deformation and brittle fracture are more likely observed on worn pressable disilicate surfaces than on unworn ones [23,24].

By comparing the qualitative spectroscopy analyses relating to the zirconia sample, it was possible to see how in zone A (untreated control surface), the presence of the Zr element is below detection level, while the most common element is silicon oxide. This is due to the glazing process carried out by the dental technician after the fabrication of the ceramic restorations. This layer was clearly compromised after instrumentation (Groups B, C and D) showing a reduced percentage of silicon oxide and, on the other hand, a considerable increase in zirconium percentage. With steel curettes instrumentation (Group C), the presence of Zr rose to 33%, which is quite significative of the erosion of the surface glaze layer of the sample. The result is a rougher and more porous surface, which increases the risk of plaque retention.

However, the release of metallic components (Iron e Chromium) from steel instruments to the sample surface was higher for mechanical instrumentation (Group D). Only for the zirconia sample, group C showed a higher release of iron and chromium than piezoelectric. This result could be explained by thinking that, during instrumentation, the roughness of zirconia considerably increased. Then, a major wear of steel curettes may be possible also because of the wider contact surface of the instrument itself, the more maintained contact and the greater and continued force applied than piezoelectric tips.

Conversely, both the disilicate samples show a comparable and significantly lower degree of surface glazing erosion compared to the zirconia sample with all the instruments used, as demonstrated by the constant presence of surface silicon oxide also after instrumentation. This could be attributed to the different bonding of the silicon oxide of the glaze with zirconia and disilicate materials. Probably, the absence of a clear chemical adhesion between glaze and zirconium [24,25], and the possible phase transformation of zirconia after applying local stresses [26] by instrumentation, could justify this difference.

There are several limitations to this study: the in vitro conditions such as the absence of saliva, blood, gingiva, and the fact that instrumentation is performed directly on the surface of the material with a constant inclination, almost impossible with in vivo conditions, obviously could have influenced the results. In addition, the round shape of the samples is different than the shape of the dental crown or other indirect restorations and on this surface, instrumentations could have been performed in an unrealistic way.

Other limits are represented by the restricted number of samples and the limited types of materials tested; it is possible that testing other types of restoration materials but also other types of zirconium and lithium disilicate may lead to different results. Furthermore, an evaluation of roughness profile values of these materials is lacking, and, in future, it could be interesting to associate it with a micro-qualitative analysis.

## 4. Conclusions

The acquisition of images and qualitative analysis have shown that all the materials tested in this study have undergone significant changes of their superficial structure after instrumentation both with sonic and manual instruments.

Steel curettes demonstrated a more aggressive behavior than the sonic one across all the samples, while plastic curettes revealed a low aggressive behavior.

The disilicate samples show a comparable and significantly lower degree of surface glazing erosion compared to zirconia sample with all the instruments used.

However, more studies with more samples, and possibly in vivo conditions are required to evaluate the superficial change in new restoration materials after routine professional hygiene procedures. The continuous evolution in the field of dental materials is generating a lot of new materials for dental restorations. It is, therefore, important to evaluate their behaviors in the mouth of the patient, especially after hygiene procedures, to understand the possible advantages or disadvantages of prosthetic treatment.

## Figures and Tables

**Table 1:** Qualitative microanalyse of CAD-CAM Zirconia.

Qualitative Analysis Zirconia	O	Si	Zr	Na	Al	K	Zn	Ca	Mg	Fe
“A zone”- non treated	wt%: 56.8% σ: 0.2	wt%: 27.4% σ: 0.1	wt%: - σ: -	wt%: 7.2% σ: 0.1	wt%: 2.6% σ: 0.0	wt%: 2.9% σ: 0.0	wt%: 1.9% σ: 0.1	wt%: 1.1% σ: 0.0	wt%: 0.3% σ: 0.0	wt%: - σ: -
“B zone”-treated with plastic curettes	wt%: 46.6% σ: 0.3	wt%: 13.4% σ: 0.1	wt%: 28.6% σ: 0.2	wt%: 3.3 σ: 0.0	wt%: 1.9% σ: 0.0	wt%: 1.8% σ: 0.0	wt%: 1.2% σ: 0.1	wt%: 0.8% σ: 0.0	wt%: - σ: -	wt%: 1.5% σ: 0.1
“C zone”-treated with SS curettes	wt%: 48.7% σ: 0.2	wt%: 8.2% σ: 0.1	wt%: 33.2% σ: 0.2	wt%: 2.4% σ: 0.1	wt%: 1.4% σ: 0.0	wt%: 0.9% σ: 0.0	wt%: 0.6% σ: 0.1	wt%: 0.4% σ: 0.0	wt%: - σ: -	wt%: - σ:-
“D zone”- treated with piezoelectric	wt%: 54.7% σ:0.2	wt%: 18.8% σ: 0.1	wt%: 13.9% σ: 0.2	wt%: 4.9% σ: 0.1	wt%: 2.3% σ: 0.0	wt%: 2.1% σ: 0.0	wt%: 1.2% σ: 0.1	wt%: 0.8% σ:0.0	wt%: 0.2% σ: 0.0	wt%: 1.1% σ: 0.1

**Table 2:** Pressed Lithium Disilicate.

Qualitative Analysis Cad-Cam Disilicate	O	Si	Na	Al	K	Zn	Ca	Mg	Ce	Fe	P	Cr
“A zone” - non treated	wt%: 64.2% σ: 0.2	wt%: 24.7% σ: 0.1	wt%: 3.3% σ: 0.1	wt%: 2.5% σ: 0.0	wt%: 3.5% σ: 0.0	wt%: 0.7% σ: 0.1	wt%: 0.9% σ: 0.0	wt%: 0.3% σ: 0.0	wt%: - σ: -	wt%: - σ: -	wt%: - σ: -	wt%: - σ: -
“B zone”-treated with plastic curettes	wt%: 55.7% σ: 0.2	wt%: 29.0% σ: 0.2	wt%: 2.4% σ: 0.1	wt%: 2.7% σ: 0.1	wt%: 5.7% σ: 0.1	wt%: 2.3% σ: 0.1	wt%: 1.2 % σ: 0.0	wt%: 0.5% σ: 0.0	wt%: 0.5% σ: 0.0	wt%: - σ: -	wt%: - σ: -	wt%: - σ: -
“C zone”-treated with SS curettes	wt%: 53.8% σ: 0.2	wt%: 28.9% σ: 0.2	wt%: 1.6% σ: 0.1	wt%: 2.4% σ: 0.1	wt%: 4.6% σ: 0.1	wt%: 1.0% σ: 0.1	wt%: 0.9% σ: 0.0	wt%: 1.4% σ: 0.0	wt%: 0.8% σ: 0.1	wt%: 2.8% σ: 0.1	wt%: 0.5% σ: 0.0	wt%: 0.5% σ: 0.1
“D zone”- treated with piezoelectric	wt%: 63.3% σ: 0.2	wt%: 24.7% σ: 0.1	wt%: 1.5% σ: 0.1	wt%: 2.2% σ: 0.0	wt%: 2.8% σ: 0.0	wt%: - σ: -	wt%: 0.4% σ: 0.0	wt%: - σ: -	wt%: - σ: -	wt%: 3.6% σ: 0.1	wt%: 0.8% σ: 0.0	wt%: 0.7% σ: 0.0

**Table 3:** CAD-CAM Lithium Disilicate.

Qualitative Analysis Pressed Lithium Disilicate	O	Si	Na	Al	K	Zn	Ca	Mg	Ce	Fe	Cr	P
“A zone” - non treated	wt%: 55.0% σ: 0.2	wt%: 29.7% σ: 0.1	wt%: 2.4% σ: 0.1	wt%: 2.8% σ: 0.0	wt%: 6.1% σ: 0.1	wt%: 2.6% σ: 0.1	wt%: 1.1% σ: 0.0	wt%: 0.4% σ: 0.0	wt%: - σ: -	wt%: - σ: -	wt%: - σ: -	wt%: - σ: -
“B zone”-treated with plastic curettes	wt%: 55.7% σ: 0.2	wt%: 30.3% σ: 0.2	wt%: 2.2% σ: 0.1	wt%: 2.6% σ: 0.0	wt%: 6.1% σ: 0.1	wt%: 3.6% σ: 0.1	wt%: 1.1 % σ: 0.0	wt%: 0.5% σ: 0.1	wt%: 0.5% σ: 0.0	wt%: 0.3% σ: 0.01	wt%: - σ: -	wt%: - σ: -
“C zone”-treated with SS curettes	wt%: 57.4% σ: 0.2	wt%: 28.7% σ: 0.1	wt%: 2.0% σ: 0.1	wt%: 2.6% σ: 0.0	wt%: 5.5% σ: 0.1	wt%: 2.7% σ: 0.1	wt%: 0.9% σ: 0.0	wt%: - σ: -	wt%: - σ: -	wt%: 0.3% σ: 0.0	wt%: - σ: -	wt%: - σ: -
“D zone”- treated with piezoelectric	wt%: 57.5% σ: 0.2	wt%: 18.8% σ: 0.1	wt%: 1.8% σ: 0.1	wt%: 2.3% σ: 0.0	wt%: 5.4% σ: 0.1	wt%: 3.0% σ: 0.1	wt%: 0.8% σ: 0.0	wt%: 0.5% σ: 0.0	wt%: - σ: -	wt%: 0.9% σ: 0.1	wt%: 0.1% σ: 0.0	wt%: 0.4% σ: 0.0

## Data Availability

The data presented in this study are available on request from the corresponding author.
